# Bilateral spondylolysis of inferior articular processes of the fourth lumbar vertebra: a case report

**DOI:** 10.3109/03009734.2011.629750

**Published:** 2012-02-15

**Authors:** Tomoaki Koakutsu, Naoki Morozumi, Takeshi Hoshikawa, Shinji Ogawa, Yushin Ishii, Eiji Itoi

**Affiliations:** ^1^Department of Orthopaedic Surgery, Nishitaga National Hospital, Sendai, Japan; ^2^Department of Orthopaedic Surgery, Tohoku University Hospital, Sendai, Japan

**Keywords:** Articular process, scoliosis, spondylolysis, surgery

## Abstract

Lumbar spondylolysis, a well known cause of low back pain, usually affects the pars interarticularis of a lower lumbar vertebra and rarely involves the articular processes. We report a rare case of bilateral spondylolysis of inferior articular processes of L4 vertebra that caused spinal canal stenosis with a significant segmental instability at L4/5 and scoliosis. A 31-year-old male who had suffered from low back pain since he was a teenager presented with numbness of the right lower leg and scoliosis. Plain X-rays revealed bilateral spondylolysis of inferior articular processes of L4, anterolisthesis of the L4 vertebral body, and right lateral wedging of the L4/5 disc with compensatory scoliosis in the cephalad portion of the spine. MR images revealed spinal canal stenosis at the L4/5 disc level. Posterior lumbar interbody fusion of the L4/5 was performed, and his symptoms were relieved.

## Introduction

One of the major causes of lumbar spondylolysis in adolescents is stress fracture during sports activities (1,2). Spondylolysis usually affects the pars interarticularis of a lower lumbar vertebra (3,4) and rarely involves the articular processes. We report a case of bilateral spondylolysis of inferior articular processes of the fourth lumbar vertebra (L4) that caused spinal canal stenosis with a significant segmental instability at L4/5 and scoliosis, successfully treated by posterior lumbar interbody fusion (PLIF).

## Case report

The case involved a 31-year-old male construction worker, whose major complaints were low back pain, scoliosis, and numbness of the right leg. His past illness and family history were non-contributory. His sports history included playing baseball in junior high school and handball in senior high school.

### Present illness

He had often had low back pain since he was a teenager. At the age of 30 years, his low back pain worsened, and he began walking with his trunk shifted to the right, with numbness of the lateral and posterior sides of the right leg. Since conservative treatment at a local clinic had proven ineffective, he was referred to our department and hospitalized for surgery.

### Clinical findings at hospitalization

**Figure 1.**
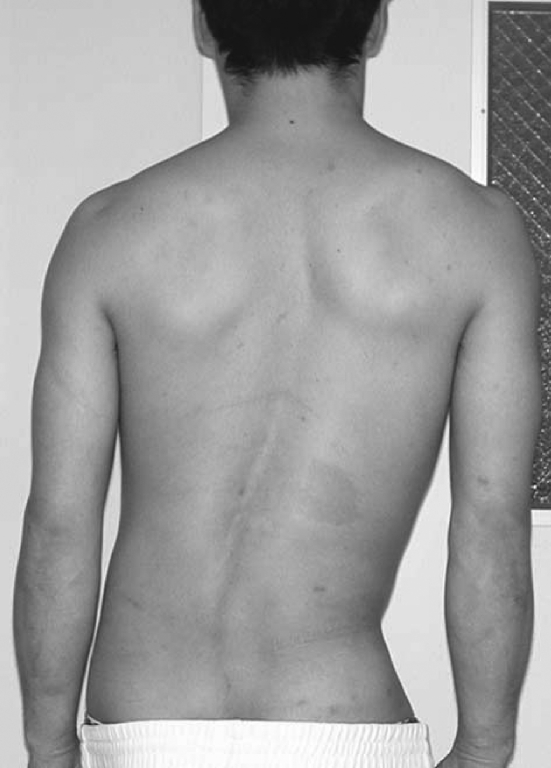
Preoperative clinical photography revealed significant scoliosis.

Significant scoliosis was present. A perpendicular line from the C7 spinal process passed through a point 5 cm to the right of the gluteal cleft (Figure 1). Numbness of the right lower leg was absent at rest but developed after a 10-minute walk. He could walk for 1 km without interruption and had no impairment of urination. On the basis of neurological examination, he was diagnosed with right L5 radiculopathy and cauda equina syndrome. The severity of his symptoms, measured using the criteria for evaluation of low back pain proposed by the Japanese Orthopaedic Association (JOA score), was 20.

### Imaging findings

**Figure 2.**
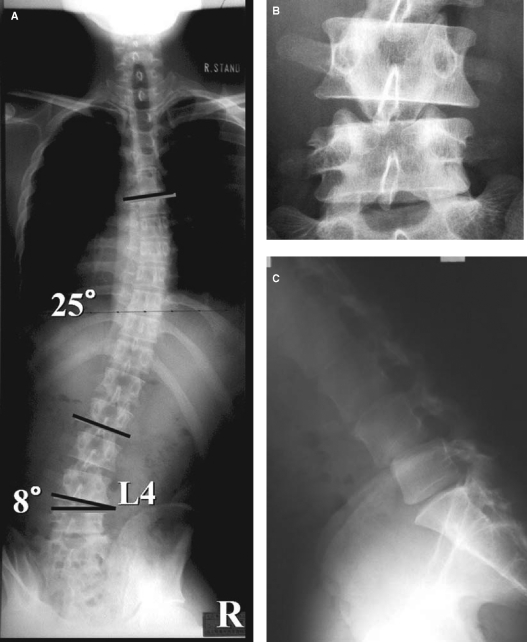
Preoperative plain X-rays. A: Posteroanterior view of the full spine in upright position revealed wedging of the L4/5 disc and the compensatory scoliosis in the cephalad portion of the spine. B: Posteroanterior view of the lumbar spine revealed bilateral spondylolysis of the inferior articular processes of the fourth lumbar vertebra. C: A lateral view of the spine revealed anterolisthesis of the L4 vertebra and a significant pathological opening of the posterior disc space at L4/5 in flexion position.

**Figure 3.**
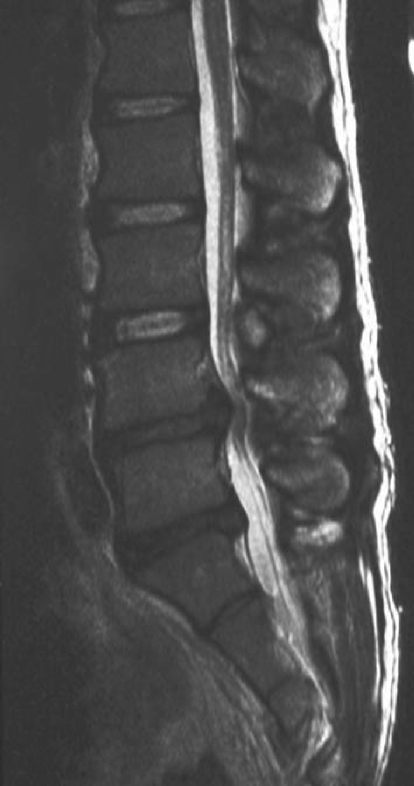
MR images of the lumbar spine revealed spinal canal stenosis at L4/5 (T2-weighted image, sagittal section).

**Figure 4.**
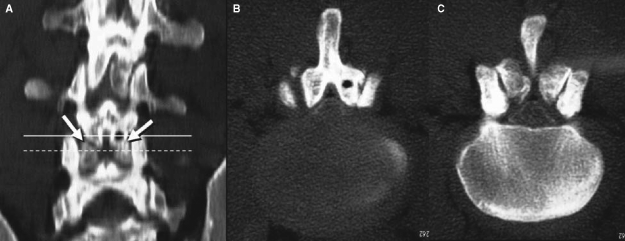
Contrast-enhanced spinal CT scan. A: Frontal plane reconstruction clearly demonstrated spondylolysis of the bilateral inferior articular processes of the L4 vertebra (arrows). B: Horizontal sections at L4/5 disc revealed sagittalization of the L4/5 facet, and deviation of the inferior articular processes of L4 to the right and anterior direction which compressed the dural sac. C: Horizontal sections at the superior margin of the L5 vertebra revealed bone fragments separated from the inferior articular processes of L4.

A plain posteroanterior view of the full spine in upright position revealed 8-degree right-sided wedging of the L4/5 disc and a 25-degree right-sided compensatory scoliosis in the cephalad portion of the spine (Figure 2A). A plain posteroanterior view of the lumbar spine revealed bilateral spondylolysis of inferior articular processes of the L4 vertebra (Figure 2B). A lateral view of the spine revealed anterolisthesis of the L4 vertebra (Meyerding grade I). There was dilation of the posterior aspect of the L4/5 disc in flexion position (Figure 2C). MR images of the lumbar spine revealed spinal canal stenosis at L4/5 (Figure 3). Contrast-enhanced spinal CT scan with frontal plane reconstruction clearly demonstrated separation of the bilateral inferior articular processes of the L4 vertebra (Figure 4A). Horizontal sections at L4/5 disc level revealed sagittalization of the L4/5 facet and deviation of the inferior articular processes of L4 to the right and anterior direction, which compressed the dural sac (Figure 4B). At the level of the superior margin of the L5 vertebra, separate bone fragments which might have been separated from the adjacent inferior articular processes of L4 were observed (Figure 4C).

### Surgery

PLIF using then Brantigan I/F cages (DePuy AcroMed Corp., Raynham, MA) and the Steffee VSP plate system (DePuy AcroMed Corp.). was performed at L4/5. Adequate decompression was achieved by removing the lower portion of the lamina of L4, the separate bone fragments, and inferior articular processes of L4. Then the Brantigan I/F cages were implanted in the L4/5 disc space, and bone allografts made of removed bone chips were attached to the outside of the cage.

### Postoperative course

**Figure 5.**
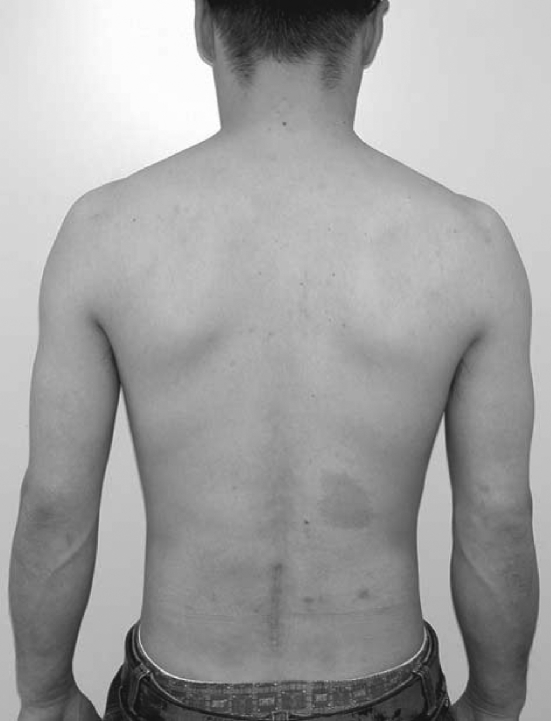
Postoperative clinical photography revealed improvement of scoliosis.

**Figure 6.**
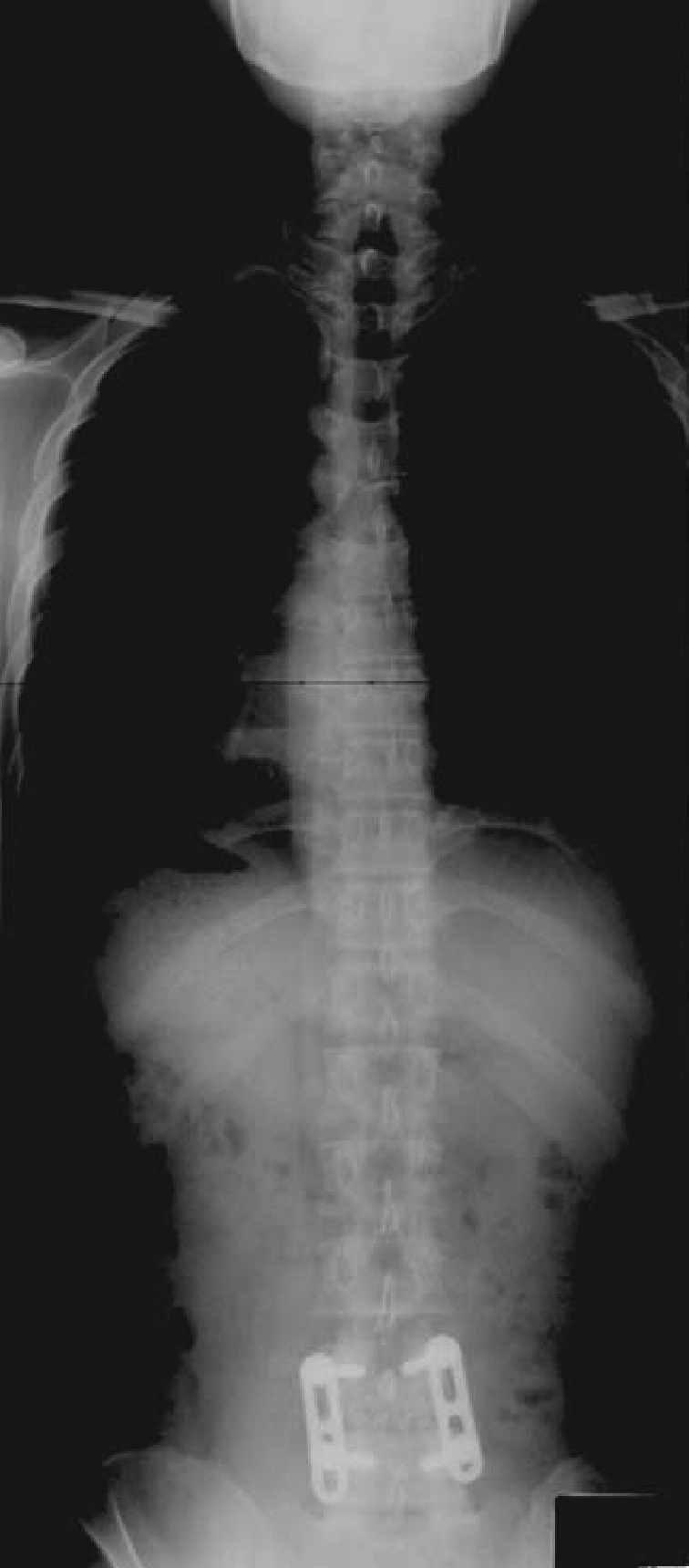
Postoperative plain posteroanterior view of the full spine in upright position revealed bony fusion and improvement of scoliosis.

After removal of the drain, the patient was allowed to sit with a hard brace. He was instructed to wear the hard brace for 3 months. On follow-up examination at 1 year after surgery, he had returned to his previous work with marked improvement of his low back pain and disappearance of scoliosis and numbness of the right lower leg (Figure 5). The follow-up X-rays revealed bony fusion and improvement of scoliosis (Figure 6). JOA score was improved to 26.

## Discussion

Lumbar spondylolysis, a well known cause of low back pain in adolescents, is often caused by fatigue fracture of the lower lumbar spine, usually at the pars interarticularis, during sports activities (1,2). There have been sporadic reports of atypical lumbar spondylolysis at the junction between the pedicle and vertebral body, at the pedicle, and at the lamina posterior to the pars interarticularis (3). Many of these cases were related to congenital incomplete ossification and injuries and differ in nature from cases of lumbar spondylolysis typically observed in adolescents.

Few reports have been made on cases of fatigue fracture and/or spondylolysis in areas other than the pars interarticularis of the lumbar vertebrae. Tanaka et al. reported a rare case of spondylolysis that traversed the lamina (4), and Ireland et al. reported a case of bilateral pedicle stress fractures in a ballet dancer (5). Cases of spondylolysis at articular processes other than those related to injuries (6) and iatrogenic cases (7) are very rare: to our knowledge, there have been only three reports of spondylolysis at the articular processes (8-10). Table I presents a summary of these three cases and our own case. All four patients were athletes during adolescence, and our patient began having low back pain during adolescence when he engaged in sports activities. Since imaging in our patient revealed that the articular processes of L4, including the separate bone fragments, were nearly normal in size, it is unlikely that he had congenital hypoplasia of the vertebral articular processes. As in the three cases reported previously, the spondylolysis in the present case appeared to have been caused by pseudoarthrosis due to fatigue fracture.

**Table I.** Reported cases of spondylolysis at articular processes in the lumbar spine.

Authors (year)	Age (years)/ Gender	Sports history	Location	Treatment
Omar (1979)	21/ F	Skiing	Left L5 SAP	Conservative
Fehlandt (1993)	36/ F	Ballet dancing	Left L4 IAP	Operation^a^
McCormack (1999)	12/ F	Gymnastics	Left S1 SAP	Conservative
This case	31/ M	Baseball, handball	Bilateral L4 IAP	Operation^b^

^a^Excise the fragment.

^b^Posterior lumbar interbody fusion (PLIF).

IAP = inferior articular process; SAP = superior articular process.

The three previously reported patients had unilateral spondylolysis, while the present patient had bilateral spondylolysis: this difference in type of spondylolysis appeared to explain why the symptoms of the three previous patients were limited to low back pain not associated with neurological abnormalities, while the present patient had degeneration of the L4/5 disc, significant instability of the spine, scoliosis, and neurological symptoms. In this case, sagittalization of the L4/5 facet (Figure 4B) is considered to have been involved in anterolisthesis. This mechanism is similar to that in lumbar degenerative spondylolisthesis. The tips of the separate inferior articular processes are not displaced to the anterior direction but remain in the posterior direction (Figure 4C). Accordingly, in addition to the instability of posterior lumbar elements caused by spondylolysis at bilateral articular processes, sagittalization of the facet may have facilitated degeneration of the intervertebral disc and resulted in marked instability of the spine in both the sagittal and frontal planes.

The present patient required spinal fusion because the affected facet joint could not be conserved when sufficient decompression was obtained. It appeared that the wedging of the L4/5 disc was a major cause of the deformation due to scoliosis, which developed even though his low back pain was mild at rest. This deformation disappeared after PLIF.
